# Supportive use of digital technologies during transition to adult healthcare for young people with long-term conditions, focusing on Type 1 diabetes mellitus: A scoping review

**DOI:** 10.1177/13674935231184919

**Published:** 2023-06-30

**Authors:** Joy Orpin, Alison Rodriguez, Deborah Harrop, Elizabeth Mills, Fiona Campbell, Jacqueline Martin-Kerry, James Turner, Janet Horsman, Jon Painter, Maddie Julian, Paul Dimitri, Philippa Howsley, Veronica Swallow

**Affiliations:** 1 7314 Sheffield Hallam University, Sheffield, UK; 2 4468 University of Leeds, Leeds, UK; 3 7315 University of Sheffield, Sheffield, UK; 4 4472 Leeds Teaching Hospitals NHS Trust, Leeds, UK; 5 4488 University of Leicester, Leicester, UK; 6 Digibete CIC, Leeds, UK; 7 7316 Sheffield Children’s NHS Foundation Trust, Sheffield, UK

**Keywords:** digital technology, chronic disease, transition to adult care, diabetes mellitus, Type 1, adolescent and young adult

## Abstract

Type 1 diabetes mellitus (T1DM) is the second most common chronic or long-term condition (LTC) affecting young people (YP); when transitioning from paediatric to adult healthcare, young people with LTCs such as T1DM are expected to self-manage medication, diet and clinical appointments. This scoping review aimed to analyse research examining ways digital health technologies were used to support YP with LTCs during transition from paediatric to adult healthcare and to establish YP’s needs, experiences and challenges when transitioning. We aimed to identify knowledge gaps and inform development of a novel chatbot with components such as avatars and linked videos to help YP with T1DM gain self-management confidence and competence during transition. Nineteen studies identified through searching five electronic databases were included in this review. A combination of digital health technologies was used to support transition of YP with LTCs to adult healthcare. Barriers to successful transition were reported and YP described the importance of social relationships and transition readiness and expressed the need for individualised interventions that acknowledge social factors such as work and college. No supportive chatbots with components to help YP with T1DM were identified. This contribution will inform future development and evaluation of such a chatbot.

## Introduction

Approximately 15 million people in England have a long-term condition (LTC). Care for people with LTCs is estimated to take up around £7 in every £10 of total health and social care expenditure in the United Kingdom (UK) (Department of Health and Social Care, 2015). There is no cure for LTCs, but patients with LTCs can manage such conditions with medication or other forms of therapy, for example, self-management support. LTCs such as type 1 diabetes mellitus (T1DM), asthma and epilepsy often develop during childhood and continue into adulthood (Department of Health, 2012). LTCs can have a detrimental physical, psychosocial and mental health impact on YP and their families, especially as they struggle to balance management of and stigma related to their condition with daily activities (Bronner et al., 2020; Wilson and Stock, 2019).

One of the significant challenges in supporting young people with LTCs is when the responsibility for healthcare is passed from a child-centred to an adult-oriented healthcare provider (Colver et al., 2019). Transition is defined as a purposeful, planned process that addresses the medical, psychosocial, educational and vocational needs of YP with LTCs as they move from child-centred to adult-oriented healthcare systems (Care Quality Commission, 2014; Department of Health, 2006; NICE, 2016). The transition process should begin in early adolescence, involve the YP, be developmentally appropriate, holistic, and be directed towards self-management and promotion of self-efficacy (Care Quality Commission, 2014; Colver et al., 2018), thus ensuring that YP are equipped with the skills and information needed to successfully self-manage their condition (NICE, 2016; NHS England, 2016).

For many YP with LTCs, transition from paediatric to adult care can be challenging, and around 50% report inadequate support and services during their transition (McManus et al., 2013; Stinson et al., 2014; Toomey et al., 2013). As an example, YP with T1DM report difficulties during Transition Clinic appointments and express dissatisfaction with the transition services (Archibald and Ashford, 2018; Hanna and Woodward, 2013).

Poor transition of YP with T1DM leads to significant health consequences due to poor clinic attendance, poor glycaemic control, disengagement from health services, and deteriorating physical and mental health (Leavey et al., 2019; Lotstein et al., 2013; Sheehan et al., 2015; Singh et al., 2010). Blood glucose control in T1DM substantially declines amongst YP aged 18–30 years, with only 14% meeting the required level (Miller et al., 2015), leading to nearly one-third of YP having evidence of early diabetes-related complications by the time of transition and a 2.5-fold elevated risk of poor glycaemic control by their first adult-service visit (Dabelea et al., 2017). Critically, poorly controlled T1DM can have acute life-threatening complications such as diabetic ketoacidosis (DKA) (Morris et al., 1997) and life-disabling chronic complications, including both micro- and macro-vascular disease (Demirel et al., 2013). The highest rates of DKA are seen in YP aged 15–20 years during transition (Karges et al., 2015).

Well-planned transition can improve educational, clinical and social outcomes for YP (Campbell et al., 2016; Crowley et al., 2011; Dogba et al., 2014). Transition interventions that focus on collaboration between YP and healthcare teams, patient education, and specific transition clinics, and that have a solid theoretical approach and include a partnership with parents, can improve transition readiness in YP, support successful transition and improve post-transition outcomes (Campbell et al., 2016; Crowley et al., 2011; Dogba et al., 2014). In YP with T1DM, support throughout transition results in improved glycaemic control, increased clinic attendance, fewer episodes of hospitalisation, lower rates of hypoglycaemia, better self-care and self-management, and increased knowledge of T1DM (Sheehan et al., 2015).

Digital interventions and support have potential to provide YP with holistic and easily accessible medical, educational/vocational, and psychosocial support that are not available or accessible during conventional clinic appointments (Gentles et al., 2010; Sutcliffe et al., 2011). During transition, YP find that the internet provides access to informational support at convenient times enabling them to maintain a high level of autonomy and anonymity, reducing feelings of isolation, reducing embarrassment about knowledge gaps and increasing their sense of autonomy (Rasmussen et al., 2007, 2011). However, digital interventions are not always evidence-based, disease specific, informed by the needs and preferences of the end-users (Majeed-Ariss et al., 2015), may be unsuitable for older adolescents and young adults, and do not deliver relevant information in an interactive, developmentally appropriate way.

In the last decade, there has been an exponential rise in the use of digital health technologies and medical devices in healthcare systems. Chatbots, for example, can be incorporated into clinical practice to improve patient outcomes (Xu et al., 2021) and have been reported to provide young people with timely mental health support (Ludin et al., 2022). Given the importance of effective transition for YP with LTCs and its impact on health outcomes in adult healthcare, there is a need to understand the currently available technologies to support YP during this process. To our knowledge, no scoping or systematic review was undertaken to synthesise the evidence on supportive use of digital health technologies through transition to adult healthcare for YP with LTCs, with a focus on T1DM.

This review aimed to synthesise literature on the supportive use of digital health technologies through transition to adult healthcare for young people with LTCs, with a focus on T1DM to inform development of a novel chatbot including components such as avatars and linked videos to help YP with T1DM gain self-management confidence during transition.

**Review aim:** To inform development of a chatbot to support successful transition to adult care of YP with T1DM.

### Review objectives

1. To analyse ways in which digital technology has been used to support YP with LTCs during their transition from paediatric to adult services.
2. To analyse ways in which digital technology was used to support YP with T1DM during transition to adult services.
3. To establish needs, experiences and challenges of YP with T1DM transitioning from paediatric to adult services.

## Method

Our a priori protocol for this scoping review followed the Arksey and O'Malley (2005) framework, and in line with this method, we did not include quality appraisals (Tricco et al., 2018).

### Search strategy

An Information Scientist led a search strategy that was developed in MEDLINE, using a combination of standardised indexed search terms and free-text terms that related to key aspects of the research aims; these were then adapted for use in other electronic databases to inform the final search strategy (see Supplementary materials Table S1).

We searched five electronic databases in February 2021: Cochrane Central Register of Controlled Trials (CENTRAL) (Wiley), CINAHL (EBSCO), MEDLINE (EBSCO), PsycINFO (ProQuest) and Scopus (Elsevier) to identify relevant studies published in English with no restriction on the publication date and country. Additionally, author, citation and reference lists of key papers were searched to identify relevant primary studies (Schlosser et al., 2006). Grey literature was not sought.

### Eligibility criteria

The studies included in this review met the inclusion criteria outlined in Table 1.

**Table 1.** Eligibility criteria.

**Population:** YP with LTCs (Objective 1) OR YP with T1DM (Objectives 2 and 3) YP were defined as those between 11–25 years
**Context:** YP in outpatient settings
**Intervention:** Any digital tools, devices, systems or resources used to support transition of YP with LTCs from paediatric to adult services. (Objectives 1 and 2)
**OR**
**Concept:** Views of YP, their families and healthcare professionals (HCPs) on needs, expectations, experiences, challenges and barriers to transition from paediatric to adult services (Objective 3)
**Study type:** Primary research only.
**Year of publication:** Studies published during or after 2015

### Study selection

Identified records were transferred to RefWorks, a web-based reference management software and de-duplication undertaken. Pilot screening of title and abstracts was undertaken by three authors, and full text by two authors to check inter-rater reliability. Three authors independently screened titles and abstracts. Full texts of remaining and potentially eligible records were retrieved and independently assessed for eligibility by six authors with a 10% independent assessment undertaken by one author. To determine the inter-rater reliability, Cohen’s kappa was calculated. Differences in screening decisions or lack of clarity about eligibility of a paper were reassessed by two authors and a shared consensus reached.

### Data extraction

Two authors piloted the data extraction template, and together with another four authors independently conducted data extraction. One author independently undertook a 10% replication. Discrepancies were resolved by discussion among authors. Extracted data included author/s; year of publication; study setting; study population, participant demographics and baseline characteristics; study aims/objectives; details of the intervention and control conditions (where appropriate); study methodology; recruitment and study completion rates; outcomes and timepoint of measurement; data collection and analysis; indicators of acceptability to users; suggested mechanisms of intervention action; YP’s needs, expectations, experiences, challenges, barriers to transitions to adult care; and patient and public involvement (PPI) partners as advisors.

### Data analysis and synthesis

We conducted a narrative synthesis of the key findings from included studies by following an iterative approach described by Popay et al. (2006) to identify the themes relevant to our review objectives. We tabulated included studies to enable a preliminary description of the results of included studies. This facilitated identification of digital technologies, an understanding of how the interventions work and themes that describe the transition needs, experiences and challenges of YP with T1DM.

## Results

### Study selection

**Figure 1.**
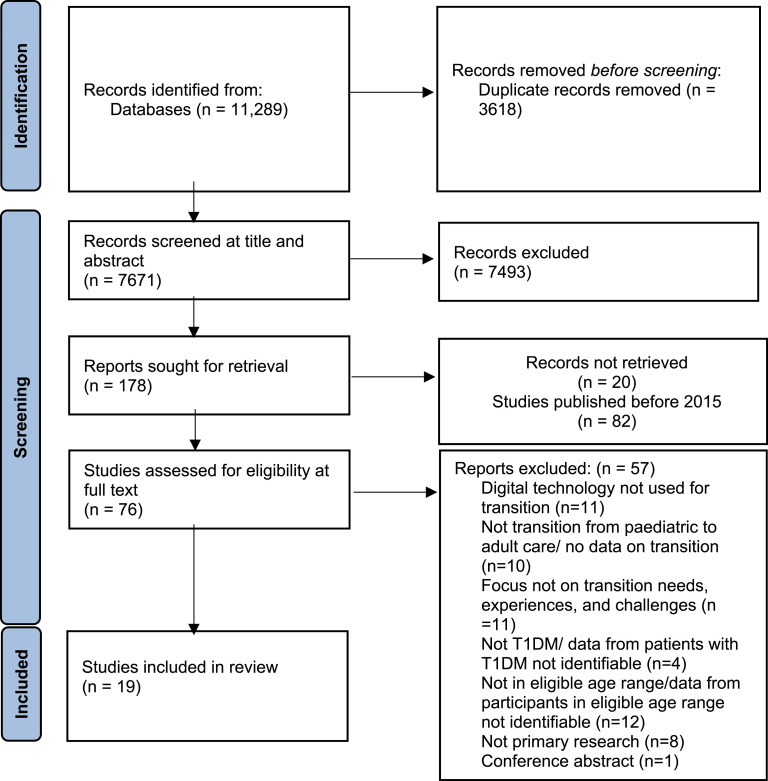
Preferred reporting items for systematic reviews and meta-analyses (PRISMA) flow diagram.

Electronic searches identified a total of 7671 records after removing duplicates. Following title and abstract screening, 7492 studies were excluded. Due to the review focusing on contemporary technology, we applied a publication date restriction and excluded 82 studies published before 2015 (NICE, 2016; OFCOM, 2015). After screening for eligibility at full text stage, 19 studies were included. See Figure 1 for the Preferred Reporting Items for Systematic reviews and Meta-Analyses (PRISMA) flow diagram. There was a moderate inter-rater agreement rate of 87.5% for the eight papers that were double screened (Cohen’s kappa = 0.6).

### Study characteristics

Included studies were published between 2015 and 2021 and conducted in the United States (*n* = 10, 53%), Canada (*n* = 4, 21%), and one study each in Norway, Ireland, Denmark, South Korea and the UK. See Table 2 for a summary of characteristics of included studies. A summary of data extracted is available as Supplementary Material (Tables S2 and S3).

**Table 2.** Summary of the 19 studies included in the review.

Lead author and year study published	Study methodology	Country of study	Age range and LTC of YP eligible, or HCP category	Sample size	Mean age years (YP)	Study participants female (%)	Patient/families as research project advisors?
Agarwal et al. (2017)	Retrospective review of medical charts [Quantitative approaches]	USA	YP (18–25 years) with T1DM	72 YP Paediatric providers (PP) 10 Adult providers (AP) 7	20.2	36 (50)	No
Albanese-O'Neill et al. (2018)	Multi-method quantitative descriptive study [Quantitative approaches]	USA	YP (18–25 years) with T1DM	20	19.2	16 (80)	No
Beaudry et al. (2019)	Quantitative descriptive study; Pre and post surveys and workshop [Mixed methods]	USA	YP (14–17 years) with LTCs	13	15.7	Not stated	Yes
Butalia et al. (2020)	Qualitative descriptive study	Canada	YP with T1DM (15–25 years) and their parents	YP 11 Parents 3	Median 18	6 (55)	No
Butalia et al. (2021)	Pragmatic non-randomised clinical trial [Non-randomised clinical trial]	Canada	YP (17–18 years) with T1DM	203 YP Usual care (UC) 102 Intervention (I) 101	UC 18.6 I 18.0	UC 53 (52) I 48 (48)	Yes
Colver et al. (2018)	Longitudinal, mixed methods [Mixed methods]	UK	YP (14–18 years) with LTCs and a parent or carer	374	*16.2	155 (41)	No
Coyne et al. (2016)	Participatory action research [Multi-methods]	Ireland	YP (14–25 years) with LTCs	207 (questionnaires) 21 (interviews) 5 Co-design 4 telephone interviews 12 participatory workshops	Data requested	Co-design 3 (60)	Yes
Garvey et al. (2017)	[Quantitative approach]	USA	YP (18–29 years) with T1DM	602 PP 303 AP 299	PP 20 AP 24	PP 182 (60) AP 186 (62)	No
Gorter et al. (2015)	Mixed-method prospective cohort study [Mixed methods]	Canada	YP in last year of paediatric care with LTCs and disabilities	50	17.9	87 (58)	No
Gray et al. (2021)	A pilot, prospective, non-randomised, intervention study [Quantitative approaches]	USA	YP (16–23 years) with IBD and their parents	36	17.4	18 (50)	Yes
Husted et al. (2018)	Qualitative exploratory study embedded in a 12-month RCT	Denmark	YP (15–23 years) with T1DM	20	18.0	11 (55)	Yes
Iversen et al. (2019)	Qualitative explorative design	Norway	YP post-transition with T1DM	11	**20.8	5 (46)	No
Kim et al. (2019)	Descriptive cross-sectional study [Quantitative approaches]	South Korea	YP (16–24 years) with T1DM	87	19.7	50 (58)	No
Leung et al. (2020)	Qualitative approach	Canada	YP (16–18 years) with T1DM	22	17.5	13 (59)	No
Lopez et al. (2018)	[Multi-methods]	USA	YP (15–21 years) with CHD	402	Median 16	169 (42)	Yes
Pierce et al. (2017)	Qualitative approach	USA	YP with T1DM recently transitioned 10; parents 9; PP 10; AP 8; HCPs or researchers 9	10	21.3	7 (70)	No
Polfuss et al. (2015)	A prospective cross-sectional study [Quantitative approaches]	USA	Families with YP with DM (16–20 years) (not yet transitioned)	45 (YP/Parent dyad) Only T1DM included in analysis	17.0	26 (58)	No
Ramchandani et al. (2019)	Qualitative descriptive study	USA	YP with T1DM (aged 18–29 years)	21	23.6	15 (71)	No
Weigensberg et al. (2018)	Non-randomised pilot trial	USA	YP with T1DM (***aged 19–25 Year)	51 37 included in analysis Intervention 9 Control 28	I 19.8 C 19.5	I 5 (56) C 13 (46)	No

*(Colver A., 2022, personal communication), **Haugstvedt A., 2022, personal communication, ***Sequeira et al. (2015).

Six studies used quantitative methods including surveys, descriptive and prospective cross-sectional designs, retrospective reviews of medical charts and pre-post interventions. Five studies were solely qualitative, and one used a qualitative method embedded in a randomised controlled trial. Three studies employed mixed methods that included a combination of qualitative, quantitative approaches and longitudinal prospective cohort studies. Two studies used multi-methods comprised of interviews, surveys, a literature reviews, participatory workshops and a co-design group. One study was a pragmatic clinical trial while another was a non-randomised trial.

Only six studies included YP, families and/or HCPs as PPI members of a project advisory group.

### Themes

Analysis resulted in three themes; each theme addresses one of the objectives.

#### How digital technology supported transition to adult services for YP with LTCs including T1DM (Objective 1)

Studies relating to this context reported using a combination of web-based applications (apps). A summary of the types of technologies used to support transition from paediatric to adult healthcare for YP and types of LTCs supported is available as Supplementary Material Table S4.

Websites contained transition information about diabetes management and technology, mental health, and a breakdown of the process of transition to adult services (Albanese-O'Neill et al., 2018; Beaudry et al., 2019; Butalia et al., 2021; Gorter et al., 2015). Structured approaches like ‘Thinking, Planning and Making’, for example, provided transition information tailored to YP’s needs and preferences to help prepare them for transition to adult healthcare (Coyne et al., 2016).

Online transition mentors, including HCPs, supported YP by offering advice on their relationships with peers, parents and general life course transitions (Gorter et al., 2015; Gray et al., 2021). Coordinators, who were not medically trained, maintained a transition website and used email, text messaging or telephone communication to answer questions from YP, notify the healthcare teams of any emergencies, maintain contact with YP following their transition to adult services and offer psychosocial support (Butalia et al., 2021).

Smartphone or mobile apps were developed to facilitate self-management and aid decision making by providing condition-specific and lifestyle information. Peer support reduced loneliness and increased safety by encouraging YP to engage with HCPs between outpatient appointments (Husted et al., 2018; Lopez et al., 2018). Apps had functionalities to remind YP about appointments, assess their treatment adherence, record mood and health status, and support preparation for clinic appointments.

Two studies reported that apps increased engagement of YP with T1DM and other LTCs, delivered educational content, improved management of the condition and developed peer support to facilitate transition (Beaudry et al., 2019; Husted et al., 2018). Beaudry et al. tested the feasibility of a text messaging platform to communicate with a healthcare professional about special health needs through the transitions of care; while Husted et al. explored the influence of a smartphone app on self-management by YP with T1DM that included a Chat Room that offered peer-to-peer interaction. Neither of these studies involved a chatbot with components to help YP with an LTC such as T1DM gain self-management confidence before and during transition

Educational videos were also used to increase YP’s knowledge and understanding, improve outcomes (e.g. psychological and engagement with clinics) and facilitate peer and social support to reduce the disconnect from their diabetes in a cost-effective way (Albanese-O'Neill et al., 2018).

The intended outcomes of digital health technology use were for a positive effect on YP’s health and support transition. The key to a successful transition process was reported to be engagement of YP, their families, and HCPs in development and use of the technologies (Coyne et al., 2016; Husted et al., 2018; Lopez et al., 2018). However, while most YP had access to smartphones, it was important to have functionality across platforms, and content and functionality needed regular updating to keep YP engaged.

#### How digital technology supported transition to adult services for YP with T1DM (Objective 2)

Only three studies reported using digital technology to support transition among YP with T1DM; these studies were in the United States (Albanese-O'Neill et al., 2018), Denmark (Husted et al., 2018) and Canada (Butalia et al., 2021) see Table S4.

YP received T1DM education through a group videoconference (Albanese-O'Neill et al., 2018). There were improvements in diabetes-related self-efficacy and distress and clinic attendance. YP found the intervention very acceptable and described its potential to improve patient-reported satisfaction, social support and attendance. In addition, using simple, readily accessible digital health technologies like text messaging and emails to enhance transition coordinator intervention, reduced a loss to follow-up in adult services (Butalia et al., 2021). Husted et al. (2018) reported that a mobile health app, ‘Young with Diabetes (YWD)’ promoted self-management through peer support that minimised loneliness and improved YP’s ability to gain skills and knowledge for managing T1DM. As a result, YP suggested the app be recommended for self-management to YP who are newly diagnosed with T1DM.

YP with T1DM found these interventions acceptable and successful in improving clinic attendance, social support (Albanese-O'Neill et al., 2018; Butalia et al., 2021) and self-management (Husted et al., 2018).

#### Transition needs, experiences and challenges of YP with T1DM (Objective 3)

Nine studies reported the needs, experiences and challenges of YP with T1DM from the perspectives of YP only (Butalia et al., 2021; Colver et al., 2018; Iversen et al., 2019; Kim et al., 2019; Leung et al., 2020; Ramchandani et al., 2019; Weigensberg et al., 2018), YP and their families (Agarwal et al., 2017; Butalia et al., 2020; Pierce et al., 2017; Polfuss et al., 2015), and HCPs (Garvey et al., 2017).

##### Needs

Individualisation of transition interventions was vital and empowered YP to regard the transition programme as relevant to their needs (Leung et al., 2020). YP reported their need for interventions to acknowledge how they manage other ‘challenges’ such as college, work, living away from home, and relationships and how these can impact their self-management of diabetes (Pierce et al., 2017; Ramchandani et al., 2019). Support from family and peers is valued. Healthcare transition readiness was positively correlated with family support and self-management competency. YP with a network of support from family and peers felt they could draw on these support systems as they face multiple life changes (Iversen et al., 2019; Kim et al., 2019).

Irrespective of readiness or family and peer support, the supporting service needs to include HCPs ready for transition action. Highly rated interventions have included good communication between paediatric and adult teams (Agarwal et al., 2017; Butalia et al., 2020; Leung et al., 2020; Pierce et al., 2017). Robust transition programmes that include, for example, ‘care navigators’ or ‘intensive patient education curricula’, can also provide support for mental health issues that can impact on self-management abilities (Garvey et al., 2017; Polfuss et al., 2015). In addition, multicomponent interventions are acceptable for YP and families where interventions include stress reduction and mindfulness techniques; they can positively influence YP’s perceptions of relatedness and continuing support (Weigensberg et al., 2018).

##### Experiences

YP described the importance of social relationships, including support from groups, peers, friends and family to share their experiences and teach them about T1DM (Butalia et al., 2020; Kim et al., 2019; Leung et al., 2020; Pierce et al., 2017; Polfuss et al., 2015; Weigensberg et al., 2018). Ongoing family involvement in T1DM care improved self-management and healthcare transition readiness (Kim et al., 2019). The ability to find an appropriate balance between YP’s autonomy and parental support and merge the management of T1DM with conflicting demands in adulthood, facilitated successful healthcare transition (Leung et al., 2020; Pierce et al., 2017). Transition support from HCPs was equally important (Garvey et al., 2017; Polfuss et al., 2015), although YP reported remaining in paediatric care because of their emotional attachment to their paediatric HCPs (Garvey et al., 2017).

Some YP received organised and developmentally appropriate interaction and counselling during transition that motivated them to continue in adult care (Agarwal et al., 2017; Colver et al., 2018; Garvey et al., 2017; Weigensberg et al., 2018). The uniqueness and ease of the transition process, compassion of the health care team and continuous involvement of YP with transition programmes increased the acceptability of the interventions (Agarwal et al., 2017). Additionally, integrative group interventions were highly acceptable and improved psychosocial wellbeing (Weigensberg et al., 2018), and the use of communication technology was helpful in self-management and enhanced clinic attendance among transitioning YP (Butalia et al., 2021).

##### Challenges

YP identified barriers to successful transition and adherence to available transition interventions. For example, delays of over 6 months before being able to make the transition were reported (Garvey et al., 2017) and once transition had occurred, coordinators did not provide medical advice, counselling or assessment of psychosocial needs (Butalia et al., 2021). Some YP reported difficulty finding adult healthcare providers who were sufficiently knowledgeable about the challenges facing YP with T1DM after transition.

There was a lack of satisfaction with the organisation of transition interventions. YP described feeling unprepared and receiving more transition information from parents than HCPs; therefore, existing routes to transfer were perceived as sub-optimal (Butalia et al., 2020; Colver et al., 2018; Iversen et al., 2019; Kim et al., 2019; Leung et al., 2020). After transition, many YP were dissatisfied with the organisation of transition programmes; there were logistical problems including difficulties travelling to clinic, limited medical advice, limited support for psychosocial needs assessment and counselling, and difficulties with healthcare insurance. Fewer adult services offered age-banded clinics, written transition plans, transition managers, or promoted self-efficacy and holistic life-skills training (Colver et al., 2018; Iversen et al., 2019; Polfuss et al., 2015; Ramchandani et al., 2019). Therefore, YP felt unprepared for the differences between paediatric and adult healthcare services and perceived they were not seen as a whole person in adult clinics, consequently, some were unable to maintain continuity of care after transition and disengaged (Iversen et al., 2019).

## Discussion

This scoping review addressed objectives 1 and 2 by identifying ways that digital health technology has been used to support the transition of YP with LTCs, with a focus on T1DM, from paediatric to adult care. The review also addressed objective 3 by highlighting the needs, experiences and challenges for YP with T1DM during transition. All included studies were from high-income countries and recruited YP with a range of LTCs from paediatric and adult healthcare settings. Few studies were specific to T1DM. Most studies that examined the transition needs, experiences and challenges of YP with T1DM involved participants aged 15 years and older.

A combination of various digital health technologies was used to support transition of YP with LTCs, although only three out of the eight studies were specific to T1DM. These technologies used various interfaces and functionalities to engage YP with LTCs and their families and facilitate transition. The LTC did not influence the type of technology selected to support the transition; hence, the digital health technologies used for YP with T1DM were like those used in other LTCs (Albanese-O'Neill et al., 2018; Beaudry et al., 2019; Husted et al., 2018; Lopez et al., 2018).

Functionalities of the digital health technologies identified in this review included interactions to enable peer support, reduce loneliness, improve mental health, enhance self-management and engagement of YP with adult services. This is in keeping with the UK National Institute for Health and Care Excellence guidelines (NICE, 2021) which recommend the use of digital health technologies, peer support, coaching and mentoring within transition-planning into adult services. The resources also need to enable YP with LTCs to easily communicate with their HCPs (Department of Health, 2011). Interestingly, this recommendation was an essential part of the technology used within the studies in this review as the digital health technologies were used by YP to enable access to HCPs between appointments and seek guidance on both health and non-health-related issues. However, previous research cautions that the use of digital communications has the potential to reduce the amount of responsibility YP take for their health due to easy access to HCPs through digital technology (Griffiths et al., 2017).

In the reviewed studies, YP expressed a preference for transition interventions that are tailored to their individual needs and recognise other social issues they may encounter such as entering the workplace, living away from home, and relationships and how these can affect self-management of T1DM. As described in a previous study (Hilliard et al., 2014), our findings reflect YP’s need for interventions that consider the distinctive challenges of such a transition period, and which engage YP in meaningful discussions about T1DM in both paediatric and adult health care settings. Therefore, our findings suggest transition programmes, including web-based and digital interventions and interactions, should be co-designed to support self-management around individual needs, empower YP and improve post-transition experience.

Consistent with our review, a recent study of YP with sickle cell disease reports participants’ preference for technologically based interventions such as phone calls, text messaging and video chats (Viola et al., 2021). Additionally, transition interventions need to consider the readiness of HCPs for the transition process. Good communication between paediatric and adult healthcare teams accounts for highly acceptable transitions (Agarwal et al., 2017; Butalia et al., 2020; Leung et al., 2020; Pierce et al., 2017). This finding is supported by evidence that highlights the significance of coordinated care between adult and paediatric diabetes teams for successful transition (Hilliard et al., 2014; Iyengar et al., 2019).

Many YP reported that social networks (support from friends, peers and family members) were significant in their transition experiences because these enabled them to share their experiences, teach others about T1DM and improve self-management and transition readiness. In line with this finding, another study with participants aged 24–35 years reported that support from parents, friends and peers with T1DM, and HCPs, is significant in the management of T1DM (McDowell et al., 2020).

Our review found that having well-organised and developmentally appropriate interactions and counselling during transition was important in motivating YP to continue in adult care. The empathy and support of the health care team and continuous involvement of YP’s services increased the acceptability of the interventions (Agarwal et al., 2017; Colver et al., 2018; Garvey et al., 2017; Polfuss et al., 2015; Weigensberg et al., 2018). Having supportive HCPs is, therefore, necessary for successful transition for YP with T1DM (McDowell et al., 2020).

Although this review found that YP with T1DM reported some positive experiences with their transition, some challenges were also identified. YP in this review were dissatisfied with the organisation of transition programmes and reported facing issues such as limited medical advice, counselling and psychosocial needs assessment. Many participants felt unprepared for the differences between paediatric and adult services and struggled to maintain continuity of care. This finding is consistent with a study (Michaud et al., 2018) that assessed HCP’s perspectives on transition, recommending that paediatric teams enhance transition readiness in YP by educating them about the differences between paediatric and adult healthcare services.

Our review identified a range of digital and web-based interventions used to support transition to adult care; however, very few studies specifically focused on YP with T1DM. Despite a recent review reporting that T1DM was the most common childhood LTC studied (Hart et al., 2019), we found no evidence for the use of digital health technologies for transition of YP with T1DM in the UK, although Beaudry et al. (2019) reported the use of a chatbot for YP in paediatric IBD, cardiology and T1DM speciality clinics in the United States and Husted et al. (2018) used a smartphone app for YP with T1DM in Denmark. Therefore, more research is needed to understand the transition needs and challenges of YP with T1DM (Hart et al., 2019; Ramchandani et al., 2019) in the UK, and the use of communication technology to support their transition (Butalia et al., 2020; Coyne et al., 2016; Leung et al., 2020).

### Strengths and limitations

This review identified digital health technologies and components used for transition of YP with T1DM and other LTCs, and the transition needs, experiences and challenges of YP with T1DM; however, none of those focusing on T1DM matched our criteria for a chatbot, and we found no evidence to inform the development of the avatar and linked videos. Nevertheless, the review results will enable us to address our future research aim: To inform development of a chatbot to support successful transition to adult care of YP with T1DM. Using a publication date restriction of 2015 increased the strength of this study because it enabled the review of recent evidence. In accordance with scoping review methodology, we did not conduct a quality appraisal of the included studies. This review considered only participants recruited from outpatient healthcare settings, which excludes YP with T1DM and other LTCs from in-patient settings.

### Implications for practice

Healthcare professionals, patients and their families are increasingly turning to health-related technologies to assist them in improving the care that is required in the management of LTCs and to improve outcomes. Despite a large volume of research in this area we found only six of the included studies mentioned involving patients as advisors in the development and design phases of these digital health technologies. Where they were not involved then clearly the opportunities for shared learning between academics, HCPs, YP and their families had been lost. In clinical practice the paradigm of care is now shifting. The NHS Long-Term Plan (NHS, 2019) states that patients and families should be involved in co-producing the plans for their care. The findings of this review highlight the need to co-design and co-deliver intervention development and evaluation studies from beginning to end with patients and families, the value of using a broad range of digital health technologies to support transition, and the need to include support for the wider social determinants of healthcare.

## Conclusion

This synthesis of primary research has investigated the ways digital health technologies have supported YP with LTCs during transition. 19 studies, all from high-income countries, were reviewed. Synthesis revealed a need for research focusing on developing interactive, developmentally appropriate, evidence-based resources, to help YP with LTCs such as T1DM gain self-management confidence before and during transition. A potential such resource is a chatbot, with components such as avatars and linked videos which can be easily accessed and tailored towards the individual needs of the patient. These should be co-produced by families, researchers, and HCPs and where possible be based on family members’ identified needs and preferences and their effectiveness be evaluated.

## Supplemental Material

Supplemental Material - Supportive use of digital technologies during transition to adult healthcare for young people with long-term conditions, focusing on Type 1 diabetes mellitus: A scoping review Supplemental Material for Supportive use of digital technologies during transition to adult healthcare for young people with long-term conditions, focusing on Type 1 diabetes mellitus: A scoping review by Joy Orpin, Alison Rodriguez, Deborah Harrop, Elizabeth Mills, Fiona Campbell, Jacqueline Martin-Kerry, James Turner, Janet Horsman, Jon Painter, Maddie Julian, Paul Dimitri, Philippa Howsley, and Veronica Swallow in Journal of Child Health Care.

Supplemental Material - Supportive use of digital technologies during transition to adult healthcare for young people with long-term conditions, focusing on Type 1 diabetes mellitus: A scoping review Supplemental Material for Supportive use of digital technologies during transition to adult healthcare for young people with long-term conditions, focusing on Type 1 diabetes mellitus: A scoping review by Joy Orpin, Alison Rodriguez, Deborah Harrop, Elizabeth Mills, Fiona Campbell, Jacqueline Martin-Kerry, James Turner, Janet Horsman, Jon Painter, Maddie Julian, Paul Dimitri, Philippa Howsley, and Veronica Swallow in Journal of Child Health Care.

Supplemental Material - Supportive use of digital technologies during transition to adult healthcare for young people with long-term conditions, focusing on Type 1 diabetes mellitus: A scoping review Supplemental Material for Supportive use of digital technologies during transition to adult healthcare for young people with long-term conditions, focusing on Type 1 diabetes mellitus: A scoping review by Joy Orpin, Alison Rodriguez, Deborah Harrop, Elizabeth Mills, Fiona Campbell, Jacqueline Martin-Kerry, James Turner, Janet Horsman, Jon Painter, Maddie Julian, Paul Dimitri, Philippa Howsley, and Veronica Swallow in Journal of Child Health Care.

## Appendix

### Abbreviations

Apps: Applications
CF: Cystic fibrosis
CHD: Congenital heart disease
DKA: Diabetic ketoacidosis
HCPs: Healthcare professionals
IBD: Inflammatory bowel disease
LTC: Long-term or chronic condition
NHS: National Health Service (UK)
NICE: National Institute for Health and Care Excellence
PPI: Patient and public involvement
PRISMA: Preferred reporting items for systematic reviews and meta-analyses
T1DM: Type 1 diabetes mellitus
YP: Young person/people
