# Supplementary material for: Supportive use of digital technologies during transition to adult healthcare for young people with long-term conditions, focusing on Type 1 diabetes mellitus: A scoping review
Source: J Child Health Care. 2023 Jun 30;29(1):204–21. doi: 10.1177/13674935231184919 (PMC11874586; doi:10.1177/13674935231184919)
Supplement: Supplemental Material - Supportive use of digital technologies during transition to adult healthcare for young people with long-term conditions, focusing on Type 1 diabetes mellitus: A scoping review [file sj-pdf-1-chc-10.1177_13674935231184919.pdf]

Supportive use of digital technologies during transition to adult healthcare for young people with long-term conditions, focusing on Type 1 diabetes mellitus: A scoping review

Supplementary material - Table S1 Terms used in MEDLINE search

Tuesday, February 09, 2021 7:07:49 AM

#	Query	Limiters/Expanders	Last Run Via	Results
S47	S5 AND S10 AND S38	Expanders - Apply equivalent subjects Search modes - Boolean/Phrase	Interface - EBSCOhost Research Databases Search Screen - Advanced Search Database - MEDLINE	1,299
S46	S5 AND S10 AND S45	Expanders - Apply equivalent subjects Search modes - Boolean/Phrase	Interface - EBSCOhost Research Databases Search Screen - Advanced Search Database - MEDLINE	655
S45	S6 OR S43 OR S44	Expanders - Apply equivalent subjects Search modes - Boolean/Phrase	Interface - EBSCOhost Research Databases Search Screen - Advanced Search Database - MEDLINE	428,465
S44	AB (diabetics OR "diabetes mellitus" OR "type 1 diabetes" OR "type I diabetes" OR "type i diabetes") OR TI ("type 1 diabetes" OR "type I diabetes" OR "type i diabetes" OR "diabetes mellitus" OR diabetics)	Expanders - Apply equivalent subjects Search modes - Boolean/Phrase	Interface - EBSCOhost Research Databases Search Screen - Advanced Search Database - MEDLINE	412,532
S43	AB diabetic OR TI diabetic	Expanders - Apply equivalent subjects Search modes - Boolean/Phrase	Interface - EBSCOhost Research Databases Search Screen - Advanced Search Database - MEDLINE	253,884
S42	AB ("moving to" OR "moving from" OR "moving on" OR "transfer to" OR transfer from" OR "transfer on" OR "moved from" OR "moved to" OR "moved on" OR "move to" OR "move from" OR "move on" OR "transferring to" OR "transferring from" OR "transferring on" OR "transferred from" OR "transferred on" OR "transferred to") OR TI ("moving to" OR "moving from" OR "moving on" OR "transfer to" OR transfer from" OR "transfer on" OR "moved from" OR "moved to" OR "moved on" OR "move to" OR "move	Expanders - Apply equivalent subjects Search modes - Boolean/Phrase	Interface - EBSCOhost Research Databases Search Screen - Advanced Search Database - MEDLINE	643,917

	from" OR "move on" OR "transferring to" OR "transferring from" OR "transferring on" OR "transferred from" OR "transferred on" OR "transferred to")			
S41	AB (video* OR game* OR blog* OR instagram OR twitter) OR TI (video* OR game* OR blog* OR instagram OR twitter)	Expanders - Apply equivalent subjects Search modes - Boolean/Phrase	Interface - EBSCOhost Research Databases Search Screen - Advanced Search Database - MEDLINE	191,594
S40	AB ("mobile device*" OR online OR phone OR ipad*) OR TI ("mobile device*" OR online OR phone OR ipad*)	Expanders - Apply equivalent subjects Search modes - Boolean/Phrase	Interface - EBSCOhost Research Databases Search Screen - Advanced Search Database - MEDLINE	158,106
S39	AB (paediatric* OR pediatric OR juvenil*) OR TI (paediatric* OR pediatric OR juvenil*)	Expanders - Apply equivalent subjects Search modes - Boolean/Phrase	Interface - EBSCOhost Research Databases Search Screen - Advanced Search Database - MEDLINE	432,903
S38	S1 OR S3 OR S8 OR S11 OR S14 OR S15 OR S16 OR S17 OR S18 OR S19 OR S20 OR S21 OR S22 OR S23 OR S24 OR S25 OR S26 OR S27 OR S28 OR S29 OR S30 OR S31 OR S32 OR S33 OR S34 OR S35 OR S36 OR S37 OR S40 OR S41	Expanders - Apply equivalent subjects Search modes - Boolean/Phrase	Interface - EBSCOhost Research Databases Search Screen - Advanced Search Database - MEDLINE	1,164,749
S37	(MH "Natural Language Processing")	Expanders - Apply equivalent subjects Search modes - Boolean/Phrase	Interface - EBSCOhost Research Databases Search Screen - Advanced Search Database - MEDLINE	4,496
S36	AB "natural language processing" OR TI "natural language processing"	Expanders - Apply equivalent subjects Search modes - Boolean/Phrase	Interface - EBSCOhost Research Databases Search Screen - Advanced Search Database - MEDLINE	3,322
S35	AB ("virtual assistant*" OR "conversational agent*" OR "conversational user interface") OR TI ("virtual assistant*" OR "conversational agent*" OR "conversational user interface")	Expanders - Apply equivalent subjects Search modes - Boolean/Phrase	Interface - EBSCOhost Research Databases Search Screen - Advanced Search Database - MEDLINE	199
S34	AB ("digital platform" OR "digital communication*") OR TI ("digital platform" OR "digital communication*")	Expanders - Apply equivalent subjects Search modes - Boolean/Phrase	Interface - EBSCOhost Research Databases Search Screen - Advanced Search Database - MEDLINE	612

S33	AB ("web application*" OR "mobile application*" OR "virtual reality" OR "social media" OR telemedicine OR telehealth) OR TI ("mobile application*" OR "virtual reality" OR "social media" OR telemedicine OR telehealth OR "web application*")	Expanders - Apply equivalent subjects<br>Search modes - Boolean/Phrase	Interface - EBSCOhost Research Databases<br>Search Screen - Advanced Search<br>Database - MEDLINE	44,633
S32	AB ("immersive reality" OR "immersive technolog*" OR internet OR computer*) OR TI ("immersive reality" OR "immersive technolog*" OR internet OR computer*)	Expanders - Apply equivalent subjects<br>Search modes - Boolean/Phrase	Interface - EBSCOhost Research Databases<br>Search Screen - Advanced Search<br>Database - MEDLINE	351,504
S31	AB ("answering service*" OR "answer service*" OR email OR e-mail OR "electronic mail") OR TI ("answering service*" OR "answer service*" OR email OR e-mail OR "electronic mail")	Expanders - Apply equivalent subjects<br>Search modes - Boolean/Phrase	Interface - EBSCOhost Research Databases<br>Search Screen - Advanced Search<br>Database - MEDLINE	15,686
S30	AB ("instant messaging" OR "mobile phone*" OR "text messag*" OR "cell phone*" OR "reminder system*") OR TI ("instant messaging OR "text messag*" OR "cell phone*" OR "reminder system*" OR "mobile phone*")	Expanders - Apply equivalent subjects<br>Search modes - Boolean/Phrase	Interface - EBSCOhost Research Databases<br>Search Screen - Advanced Search<br>Database - MEDLINE	13,875
S29	AB (mhealth OR mhealth OR "mobile health") OR TI (mhealth OR m-health OR "mobile health")	Expanders - Apply equivalent subjects<br>Search modes - Boolean/Phrase	Interface - EBSCOhost Research Databases<br>Search Screen - Advanced Search<br>Database - MEDLINE	5,773
S28	AB (app OR apps OR "digital health" OR "digital technology") OR TI (app OR apps OR "digital health" OR "digital technology")	Expanders - Apply equivalent subjects<br>Search modes - Boolean/Phrase	Interface - EBSCOhost Research Databases<br>Search Screen - Advanced Search<br>Database - MEDLINE	31,937
S27	AB ("smart media" OR smartphone* OR "video conferenc*" OR animation OR "computerdelivered" OR "videobased" OR "video based") OR TI ("smart media" OR smartphone* OR "video	Expanders - Apply equivalent subjects<br>Search modes - Boolean/Phrase	Interface - EBSCOhost Research Databases<br>Search Screen - Advanced Search<br>Database - MEDLINE	19,390

	conferenc*" OR animation OR "computerdelivered" OR "videobased" OR "video based")			
S26	(MH "Telemedicine+")	Expanders - Apply equivalent subjects<br>Search modes - Boolean/Phrase	Interface - EBSCOhost Research Databases<br>Search Screen - Advanced Search<br>Database - MEDLINE	32,360
S25	AB (robotics OR "artificial intelligence" OR "chat bot*" OR robot OR robots OR "augmented reality" OR "artificial reality") OR TI ("artificial intelligence" OR "chat bot*" OR robot OR robots OR "augmented reality" OR "artificial reality" OR robotics)	Expanders - Apply equivalent subjects<br>Search modes - Boolean/Phrase	Interface - EBSCOhost Research Databases<br>Search Screen - Advanced Search<br>Database - MEDLINE	58,623
S24	(MH "Social Media")	Expanders - Apply equivalent subjects<br>Search modes - Boolean/Phrase	Interface - EBSCOhost Research Databases<br>Search Screen - Advanced Search<br>Database - MEDLINE	9,236
S23	(MH "Computer-Assisted Instruction")	Expanders - Apply equivalent subjects<br>Search modes - Boolean/Phrase	Interface - EBSCOhost Research Databases<br>Search Screen - Advanced Search<br>Database - MEDLINE	11,991
S22	(MH "User-Computer Interface")	Expanders - Apply equivalent subjects<br>Search modes - Boolean/Phrase	Interface - EBSCOhost Research Databases<br>Search Screen - Advanced Search<br>Database - MEDLINE	37,817
S21	(MH "User-Computer Interface")	Expanders - Apply equivalent subjects<br>Search modes - Boolean/Phrase	Interface - EBSCOhost Research Databases<br>Search Screen - Advanced Search<br>Database - MEDLINE	37,817
S20	(MH "Computers, Handheld")	Expanders - Apply equivalent subjects<br>Search modes - Boolean/Phrase	Interface - EBSCOhost Research Databases<br>Search Screen - Advanced Search<br>Database - MEDLINE	3,710
S19	(MH "Microcomputers") OR (MH "Computers")	Expanders - Apply equivalent subjects<br>Search modes - Boolean/Phrase	Interface - EBSCOhost Research Databases<br>Search Screen - Advanced Search<br>Database - MEDLINE	61,946
S18	(MH "Internet") OR (MH	Expanders - Apply	Interface - EBSCOhost	75,160

	"Internet-Based Intervention")	equivalent subjects<br>Search modes - Boolean/Phrase	Research Databases<br>Search Screen - Advanced Search<br>Database - MEDLINE	
S17	(MH "Videoconferencing")	Expanders - Apply equivalent subjects<br>Search modes - Boolean/Phrase	Interface - EBSCOhost Research Databases<br>Search Screen - Advanced Search<br>Database - MEDLINE	1,767
S16	(MH "Electronic Mail")	Expanders - Apply equivalent subjects<br>Search modes - Boolean/Phrase	Interface - EBSCOhost Research Databases<br>Search Screen - Advanced Search<br>Database - MEDLINE	2,733
S16	(MH "Electronic Mail")	Expanders - Apply equivalent subjects<br>Search modes - Boolean/Phrase	Interface - EBSCOhost Research Databases<br>Search Screen - Advanced Search<br>Database - MEDLINE	2,733
S15	(MH "Cell Phone+")	Expanders - Apply equivalent subjects<br>Search modes - Boolean/Phrase	Interface - EBSCOhost Research Databases<br>Search Screen - Advanced Search<br>Database - MEDLINE	16,252
S14	(MH "Reminder Systems")	Expanders - Apply equivalent subjects<br>Search modes - Boolean/Phrase	Interface - EBSCOhost Research Databases<br>Search Screen - Advanced Search<br>Database - MEDLINE	3,559
S13	(MH "Child")	Expanders - Apply equivalent subjects<br>Search modes - Boolean/Phrase	Interface - EBSCOhost Research Databases<br>Search Screen - Advanced Search<br>Database - MEDLINE	1,716,224
S12	(MH "Young Adult")	Expanders - Apply equivalent subjects<br>Search modes - Boolean/Phrase	Interface - EBSCOhost Research Databases<br>Search Screen - Advanced Search<br>Database - MEDLINE	889,630
S11	AB (digital OR technology OR technologies or virtual or telehealth or telecommunication* or telemedicine) OR TI (digital OR technology or virtual or telehealth or telecommunication* or telemedicine OR technologies)	Expanders - Apply equivalent subjects<br>Search modes - Boolean/Phrase	Interface - EBSCOhost Research Databases<br>Search Screen - Advanced Search<br>Database - MEDLINE	629,553

S10	S7 OR S9 OR S42	Expanders - Apply equivalent subjects Search modes - Boolean/Phrase	Interface - EBSCOhost Research Databases Search Screen - Advanced Search Database - MEDLINE	425,015
S11	AB (digital OR technology OR technologies or virtual or telehealth or telecommunication* or telemedicine) OR TI (digital OR technology or virtual or telehealth or telecommunication* or telemedicine OR technologies)	Expanders - Apply equivalent subjects Search modes - Boolean/Phrase	Interface - EBSCOhost Research Databases Search Screen - Advanced Search Database - MEDLINE	629,553
S10	S7 OR S9 OR S42	Expanders - Apply equivalent subjects Search modes - Boolean/Phrase	Interface - EBSCOhost Research Databases Search Screen - Advanced Search Database - MEDLINE	425,015
S9	(MH "Transition to Adult Care")	Expanders - Apply equivalent subjects Search modes - Boolean/Phrase	Interface - EBSCOhost Research Databases Search Screen - Advanced Search Database - MEDLINE	1,527
S8	AB ("digital technolog*" or "mobile technolog*" or "screen device*") OR TI ("digital technolog*" or "mobile technolog*" or "screen device*")	Expanders - Apply equivalent subjects Search modes - Boolean/Phrase	Interface - EBSCOhost Research Databases Search Screen - Advanced Search Database - MEDLINE	4,485
S7	AB transition* OR TI transition*	Expanders - Apply equivalent subjects Search modes - Boolean/Phrase	Interface - EBSCOhost Research Databases Search Screen - Advanced Search Database - MEDLINE	424,716
S6	(MH "Diabetes Mellitus, Type 1")	Expanders - Apply equivalent subjects Search modes - Boolean/Phrase	Interface - EBSCOhost Research Databases Search Screen - Advanced Search Database - MEDLINE	76,879
S5	S2 OR S4 OR S12 OR S13 OR S39	Expanders - Apply equivalent subjects Search modes - Boolean/Phrase	Interface - EBSCOhost Research Databases Search Screen - Advanced Search Database - MEDLINE	3,143,055
S4	(MH "Adolescent")	Expanders - Apply equivalent subjects Search modes - Boolean/Phrase	Interface - EBSCOhost Research Databases Search Screen - Advanced Search Database - MEDLINE	2,064,061

S3	(MH "Artificial Intelligence")	Expanders - Apply equivalent subjects<br>Search modes - Boolean/Phrase	Interface - EBSCOhost Research Databases<br>Search Screen - Advanced Search<br>Database - MEDLINE	24,568
S2	AB ("young adult*" or adolescent* or teenager* OR "young person" OR "young person*" OR child* OR "young people" OR teen OR teens OR youth or youths OR "early adulthood") OR TI ("young adult*" or adolescent* or teenager* OR "young person" OR "young person*" OR child* OR "young people" OR teen OR teens OR youth OR youths OR "early adulthood")	Expanders - Apply equivalent subjects<br>Search modes - Boolean/Phrase	Interface - EBSCOhost Research Databases<br>Search Screen - Advanced Search<br>Database - MEDLINE	1,673,485
S1	AB (knowbot OR chatbot* or "virtual agent*" or "virtual assistant*" or bot or bots OR "artificial intelligence") OR TI (knowbot OR chatbot* or "virtual agent*" or "virtual assistant*" or bot or bots or "artificial intelligence")	Expanders - Apply equivalent subjects<br>Search modes - Boolean/Phrase	Interface - EBSCOhost Research Databases<br>Search Screen - Advanced Search<br>Database - MEDLINE	11,698
