# Supplementary material for: Supportive use of digital technologies during transition to adult healthcare for young people with long-term conditions, focusing on Type 1 diabetes mellitus: A scoping review
Source: J Child Health Care. 2023 Jun 30;29(1):204–21. doi: 10.1177/13674935231184919 (PMC11874586; doi:10.1177/13674935231184919)
Supplement: Supplemental Material - Supportive use of digital technologies during transition to adult healthcare for young people with long-term conditions, focusing on Type 1 diabetes mellitus: A scoping review [file sj-pdf-2-chc-10.1177_13674935231184919.pdf]

Supplementary Material

One study, Butalia 2021 satisfied the inclusion criteria for both use of digital technology and transition needs and is included in both tables

Table S2 Summary of the studies that used digital technologies to support transition for YP with LTCs (n=8)

Authors & Year	Study setting and country	Study population	Study methodology	Data collection and analysis methods	Details of the digital tech(s) used to support transition to adult services	Outcomes and times of measurement	Indicators of acceptability to users	Patient/families as advisors?
Albanese -O'Neill et al. (2018)	USA<br>Outpatient diabetes clinic	20 YP (18-25 years) with T1DM	Multi-method quantitative descriptive study<br>[Quantitative approaches]	Surveys<br>Analysis: descriptive Statistical analysis	5 x 30-minute group diabetes education video conferences over an 8-week period and follow-up web-based information	Measurement at baseline and at 8 weeks of diabetes-induced distress and self-efficacy. Post treatment measurement of satisfaction and supplementary webpage views. Reduction in diabetes-related distress and in diabetes-specific self-efficacy (but not statistically significant).	Videoconference was acceptable to YP with T1DM	No
Beaudry et al. (2019)	USA<br>Pediatric Inflammatory Bowel Disease, Cardiology, and Type I Diabetes specialty clinics	13 YP (14-17yrs) with LTCs	Quantitative descriptive study;<br>Pre and post surveys and workshop<br>[Mixed methods]	Surveys; semi-structured interviews with and observations	Text messaging, chatbot & educational webpages	Increased patient participation in many important activities needed for successful health care transitions, like managing medications. YP appreciated the peer-to-peer interactions of the transition of care workshop and indicated a strong interest in more opportunities to connect with YP who can provide mentorship along the transition of care journey. The workshop was helpful in teaching YP transition related topics.	Text messaging platform was feasible and well-received by patients and caregivers	Yes
Butalia et al. (2021)	Canada<br>An adult community hospital and a	YP (aged 17–18 years) with T1DM	Pragmatic non-randomised clinical trial<br>[Non-	Electronic Medical Record; National Ambulatory Care Reporting System;	Communication technology (i.e. text messaging, email, phone call)	The primary outcome was the proportion of YP that did not attend at least one routine clinic visit in adult diabetes care	Intervention was well accepted and embraced by participants	Yes

	regional diabetes centre		randomised clinical trial]	and Discharge Abstract		within 1 year after transfer. Secondary outcomes included diabetes-related clinical outcomes and quality of life measures. Secondary clinical outcomes were assessed at 12- and 18-months post transfer.		
Coyne et al. (2016)	Ireland Recruited from a previous project, and social media and voluntary organisations	YP (aged 15–25 years) with LTCs	Participatory action research [Multi methods]	Survey; interviews, participatory workshops, video recordings, two advisory groups, and a co-design group)	Online resources (SteppingUP.ie) - website and information materials	Not reported	Not reported	Yes
Gorter et al. (2015)	Canada - 2 academic paediatric hospitals (13 clinics)	50 YP (mean age 17.9±0.9 years) with a range of neurodevelopmental conditions and other LTCs	Mixed-method prospective cohort study [Mixed methods]	Questionnaire and qualitative semi structured interviews	The KIT: Keeping It Together for Youth, tool (the 'Youth KIT') and online transition mentor	Frequency of use: 85% of YP reported using the health section of the Youth KIT at least once; 20 YP engaged in chats with the mentor.<br>Utility: the perceived utility of both interventions was modest.<br>Impact: Youth KIT received the highest ratings for 'help with goal setting'.<br>Goal achievement performance and satisfaction increased over time	Participants' perceptions about the use of the Youth KIT and the online mentor were modest	No
Gray et al. (2021)	USA IBD centres	36 YP (16–20 years) with IBD and their parents	A pilot, prospective, non-randomised, intervention study [Quantitative approaches]	Questionnaire Analysis: Descriptive statistical analysis using IBM SPSS	Face-to-face group session and four individualised tele-health sessions with a transition coach	Primary outcomes were feasibility and acceptability. Secondary outcomes were changes in transition readiness, self-management skill acquisition, perceived readiness to transfer to adult care, and disease knowledge.	Participants reported liking the group + individual format and found helpful.<br>Participants gave moderate ratings for length of time	Yes

						Participant ratings for overall program satisfaction, perceived helpfulness, and program length and format were positive.	and number of sessions, suggesting that they did not consider the time commitment to be too short or too long	
Husted et al. (2018)	Denmark<br>3 paediatric and 3 adult departments	20 YP (15-23 years) with T1DM	Qualitative exploratory study embedded in a 12-month RCT	Individual interviews<br>Analysis: Six-phased thematic analysis	Smart phone App called Young with Diabetes<br>Young with Diabetes (YWD) mHealth app provides a supplemental tool to support YP in developing T1DM self-management knowledge and skills during the transition. The app use was 3 to 64 days.	Outcomes measures for the RCT included post-trial haemoglobin A1c (HbA1c) levels (primary outcome) and scores on 3 psychometric scales (secondary outcome): Perceived Competence in Diabetes, Health Care Climate Questionnaire, and Problem Areas in Diabetes	YP recommended YWD as a supplement to self-management for newly diagnosed YP with T1DM and suggested improvements in app content and functionality	Yes
Lopez et al. (2018)	USA<br>A hospital-based paediatric cardiology clinic serving patients with CHD	402 YP (15-22 years) with CHD	[Multi methods]	Interviews; Self reporting questionnaire<br>Analysis: Data from all interviews and self-report questionnaires were manually entered into a Research Electronic Data Capture database	A prototype mobile app to facilitate transition to adult care.	Not reported	Formative phase to inform development of the app. Next steps include surveying HCPs on the content in the mobile app and focus groups on the existing prototype of the app to obtain feedback on the design, interface, and information included in the	Yes

							mobile app.	

Table S3 Summary of the studies on the transition needs, experiences, challenges, and barriers for YP with T1DM (n=12)

Authors & Year	Country and Study setting	Study population	Study methodology	Data collection and analysis methods	Patient/families as advisors?	YP's needs, expectations, experiences, challenges, barriers of transitions to adult HC
Agarwal et al. (2017)	USA<br>Paediatric to Adult Diabetes Transition Clinic	72 YP (aged 18-25 years); paediatric providers (n 10) and adult providers (n = 7)	Retrospective review of medical charts [Quantitative approaches]	Electronic medical records. Analysis: Data were analysed with STATA 14.0	No	Positive responses from YP included receiving more developmentally appropriate interactions as motivation to continue in adult care. Negative responses included logistical issues related to travel but were not inclusive of care received.<br>Positive responses from paediatric providers included ease of the transfer process, uniqueness of the program, and compassion of the adult care team. Ideas for improvement included collaborative in-person conferences between the paediatric and adult care teams; Utilization of a paediatric liaison to meet with the adult team; Adult team should notify the paediatric team of issues contacting patients.<br>Positive responses from adult providers included having a passionate adult care team and continuing to engage YP in care with components of program. Ideas for improvement included more frequent visits; and Accommodating clinic slots during afternoon or evening hours that align with patients' school schedules.
Butalia et al. (2020)	Canada<br>Large Children's Hospital diabetes program	YP with T1DM (aged 15–25 years) in and their parents	Qualitative descriptive study	Focus groups<br>Analysis: Conventional Qualitative content analysis, thematic analysis	No	All participants discussed communication technology and importantly, the absence of the use of communication technology during the transfer from paediatric to adult diabetes care.<br>YP and families felt they did not receive adequate education and preparation and others perceived no preparation for transfer and transition was provided.<br>YP and parents highlighted the importance of a peer, or group of others to share experiences and provide social support.

Butalia et al. (2021)	Canada<br>An adult community hospital and a regional diabetes centre.	YP (aged 17-18 years) with T1DM	Pragmatic non-randomised clinical trial [Non-randomised clinical trial]	Electronic Medical Record; National Ambulatory Care Reporting System; and Discharge Abstract	Yes	The transition coordinator did not provide any medical advice, counselling, or assessment of psychosocial needs.
Colver et al. (2018)	UK<br>Child healthcare services in a range of locations	374 YP (aged 14-18 years) with T1DM	Longitudinal, mixed methods [Mixed methods]	A questionnaire; Interviews with family members and health professionals, along with observations of clinical consultations<br>Analysis: using chi-squared or t-tests as appropriate; and qualitative analysis	No	Overall, the nine proposed beneficial features of transition services were poorly provided. To varying degrees, YP reported that they had not experienced the features which services said they provided. From qualitative interviews and observations, there were variations in the meaning of the features transition services as experienced by YP and families was evident.
Garvey et al. (2017)	USA<br>T1DM Exchange Clinic Registry	303 YP (aged 18–30 years) with T1DM	[Quantitative approach]	Survey	No	More than 80% of respondents reported receiving counselling on T1DM self-management and screening tests from paediatric providers, but less than half reported discussing reproductive health. In the paediatric group, half had discussed transfer with paediatric providers. Of the adult participants, 63% received an adult provider referral, and 66% felt mostly/completely prepared to transition. Adult participants with fewer pre-transition paediatric visits or who felt unprepared for transition had increased odds of gaps >6 months between paediatric and adult care. Receipt of transition preparation counselling was not associated with self-reported haemoglobin in

						either group.
Iverson et al. (2019)	Norway Outpatient clinic at a large hospital	11 YP (aged 19-23 years) with T1DM	Qualitative explorative design	Data collection: Individual interviews Data analysis was guided by Interpretive Description methodology and was conducted concurrently with the data collection	No	YP with T1DM were (1) not fully satisfied with how the transition from paediatric to adult care was organised, (2) not prepared for the differences between paediatric and adult care and (3) expressed a feeling of not being seen as a whole person in the adult clinics (4) had limited expectations regarding how the health care services were organised (5) were given limited information before the transition and received information concerning the transition from their parents.
Kim et al. (2019)	South Korea An outpatient clinic and an online self-help group for emerging adults with T1DM	87 YP (aged 16–24 years) with T1DM	Descriptive cross-sectional study [Quantitative approaches]	Data collection: Surveys using self-administered questionnaires. Analysis: Statistical analysis using SPSS version 23.0.	No	Healthcare transition readiness was significantly positively correlated with family and self-management competency. Most participants reported higher transition readiness on disease knowledge and medication management, when compared to other subcategories, such as provider communication and engagement during appointments. Ongoing family involvement in diabetes management is necessary to enhance self-management competency and healthcare transition readiness. The primary factors associated with healthcare transition readiness were identified as self-management competency and age.
Leung et al. (2020)	Canada An urban academic paediatric hospital	22 YP (aged 16-18 years) with T1DM	Qualitative approach	Data collection: focus groups and a embedded a quantitative survey towards the end of the focus groups. Analysis: inductive and	No	Four themes were identified: Individualization—how to personalize the transition experience (having choices in the transition experience, meeting adult provider before transition and specific transition preparation). Identity—how the world relates to my diabetes (stigma of type 1 diabetes, confusion with type 2 diabetes, diagnosis disclosure and resilience). Interconnection—how my support system can help

				iterative process using NVivo 12 and Statistical analysis		me with my diabetes (peer support, near peer support, parental support, loss of bond with paediatric team and fear of not having a bond with adult team). Impediment—how my diabetes limits me (self-care takes work and time, unpredictability and restrictiveness, and emotional burden). Highly rated interventions from the survey included: good communication between the paediatric and adult teams, medical summary of past diabetes care, and having paediatric and adult teams in the same building.
Pierce at al. 2017	USA Paediatric and Adult T1D specialty care facilities	YP with T1DM (aged 18-25 years) (n = 10); parents (n = 9); paediatric T1DM providers (n = 10); adult T1DM (n = 8); and HCPs or researchers with expertise in healthcare transition for YP with T1DM (n = 9)	Qualitative approach	Data collection: Interviews were semi-structured, with open-ended questions. Data analysis: Direct content analysis	No	Forming a collaborative relationship required that the adult T1DM providers be willing and able to meet the YP's with T1DM's unique needs. Another component of integrating T1DM into emerging adult roles required talking about T1DM and advocating for one's needs. This included forming social relationships that were supportive of T1DM and teaching friends about T1DM. This aspect of success also included managing college and work environments to meet T1DM care needs.<br>YP who owned their disease also took responsibility for communicating their needs to others and recognizing and seeking support from parents when needed.<br>Although ideal transition was described as "seamless" and "effortless," this was not representative of most YP due to health care system barriers (e.g., difficulties with insurance, finding adult providers who were sufficiently knowledgeable about T1DM, scheduling new patient appointments, and transferring medical records).<br>Another key indicator of successfully establishing and maintaining continuity of care was when the YP anticipated barriers and demonstrated commitment to resolving them.

Polfuss et al. 2015	USA Recruited from a paediatric diabetes programme.	45 YP (aged 16 - 20 years) and their parents	A prospective cross-sectional study [Quantitative approaches]	Data collection: Questionnaire survey<br>Analysis: Statistical analysis was completed using IBM SPSS statistics version 22.	No	YP and parents rated the YP's knowledge and self-efficacy as moderate to high and rated most behaviours and skills as very important for transition and rated the YPs' self-management behaviours as high. However, they did not agree on behaviours important for transition such as, accurately counting carbohydrates, how to check ketones, how alcohol and drugs affect diabetes, or consistent documentation of blood sugar, carbohydrates, and insulin doses.<br>YP indicated talking with providers and program materials as helpful but attending regular visits and talking with parents most helpful for transition. When reviewing the perception of helpfulness of the transition classes and the educational binder, it was found that approximately 50% of the respondents had not utilized these resources.
Ramchandani et al. 2019	USA Paediatric and adult diabetes clinics of an urban academic medical centre.	21 YP with T1DM (aged 18-29 years – mean 23.6)	Qualitative descriptive study	Data collection: focus groups<br>Analysis: Qualitative content data analysis	No	The struggle between the management of T1DM and life appeared in every situational transition of emerging adulthood: college, work, living away from home, and relationships. YP desired to achieve optimal glycaemic control because it made them feel better, and they were all doing the best they could to get there. YP didn’t perform self-management tasks as they wanted to keep their diabetes private. Fear of hypoglycaemia challenged optimal glycaemic control. Diabetes technology overall made T1DM management much easier and minimised both fear of and incidence of hypoglycaemia. YP reported the need for constant attention to their T1DM, whether or not others were aware of it, and expressed the desire to have a connection with their diabetes provider. Use of insulin pumps and continuous glucose monitors and attendance at diabetes camp decreased some of the

						DSM challenges. Different groups of individuals had different perspectives on living with diabetes and different approaches to DSM.
Weigensberg et al. 2018	USA Pediatric diabetes clinics	51 YP with T1DM (*aged 19-25)	Non-randomised pilot trial	Focus groups Data analysis: pre- and post-intervention;	No	Participants in intervention group showed significant reductions in perceived stress and depression and increases in general well-being relative to other control participants. Reduction in perceived stress, independent of intervention group, was associated with reductions in haemoglobin A1C.

***From LEAP study** Sequeira PA, Pyatak EA, Weigensberg MJ, et al. Let's empower and prepare (LEAP): evaluation of a structured transition program for young adults with type 1 diabetes. Diabetes Care. 2015;38:1412–1419
