# Supplementary material for: Supportive use of digital technologies during transition to adult healthcare for young people with long-term conditions, focusing on Type 1 diabetes mellitus: A scoping review
Source: J Child Health Care. 2023 Jun 30;29(1):204–21. doi: 10.1177/13674935231184919 (PMC11874586; doi:10.1177/13674935231184919)
Supplement: Supplemental Material - Supportive use of digital technologies during transition to adult healthcare for young people with long-term conditions, focusing on Type 1 diabetes mellitus: A scoping review [file sj-pdf-3-chc-10.1177_13674935231184919.pdf]

Supplementary Material

Table S4 Digital health technologies used to support transition for YP with LTCs (n = 8, 42%)

Author	Digital health technologies used to support transition	Type of long term condition(s)
Albanese-O'Neill et al. (2018)	Group videoconference	Type 1 diabetes mellitus
Beaudry et al. (2019)	Chatbot (text messaging platform)	Paediatric Inflammatory Bowel Disease, Cardiology, and Type 1 diabetes mellitus specialty clinics
Butalia et al. (2021)	Website content<br>Text messaging<br>Email<br>Phone call	Type 1 diabetes mellitus
Coyne et al (2016)	Online resources - website and information materials	Congenital heart disease, Cystic Fibrosis and Type 1 diabetes mellitus
Gorter et al. (2015)	Online transition mentor<br>Website<br>Group chats<br>Email	Neurodevelopmental conditions and other LTCs
Gray et al (2021)	Online telehealth sessions<br>Online transition coach<br>Face to face group sessions	Inflammatory Bowel Disease
Husted et al. (2018)	Smartphone app (mHealth app)<br>ChatRoom - messaging	Type 1 diabetes mellitus
Lopez et al. (2018)	Mobile app	Congenital heart disease
